# *Trueperella pyogenes* Isolates from Livestock and European Bison (*Bison bonasus*) as a Reservoir of Tetracycline Resistance Determinants

**DOI:** 10.3390/antibiotics10040380

**Published:** 2021-04-03

**Authors:** Ewelina Kwiecień, Ilona Stefańska, Dorota Chrobak-Chmiel, Magdalena Kizerwetter-Świda, Agata Moroz, Wanda Olech, Marina Spinu, Marian Binek, Magdalena Rzewuska

**Affiliations:** 1 Department of Preclinical Sciences, Institute of Veterinary Medicine, Warsaw University of Life Sciences, Ciszewskiego 8 St., 02-786 Warsaw, Poland; ilona_stefanska@sggw.edu.pl (I.S.); dorota_chrobak@sggw.edu.pl (D.C.-C.); magdalena_kizerwetter_swida@sggw.edu.pl (M.K.-Ś.); marian_binek@sggw.edu.pl (M.B.); 2 Division of Veterinary Epidemiology and Economics, Institute of Veterinary Medicine, Warsaw University of Life Sciences, Nowoursynowska 159c St., 02-786 Warsaw, Poland; agata_moroz@sggw.edu.pl; 3 Department of Animal Genetics and Conservation, Institute of Animal Sciences, Warsaw University of Life Sciences, Ciszewskiego 8 St., 02-786 Warsaw, Poland; wanda_olech@sggw.edu.pl; 4 Department of Infectious Diseases and Preventive Medicine, Law and Ethics, University of Agricultural Sciences and Veterinary Medicine, Calea Mănăștur 3-5, 400372 Cluj-Napoca, Romania; marina.spinu@usamvcluj.ro

**Keywords:** antimicrobial resistance, European bison, livestock, tetracycline, *tet* genes, transposons, *Trueperella pyogenes*

## Abstract

Determinants of tetracycline resistance in *Trueperella pyogenes* are still poorly known. In this study, resistance to tetracycline was investigated in 114 *T. pyogenes* isolates from livestock and European bison. Tetracycline minimum inhibitory concentration (MIC) was evaluated by a microdilution method, and tetracycline resistance genes were detected by PCR. To determine variants of *tetW* and their linkage with mobile elements, sequencing analysis was performed. Among the studied isolates, 43.0% were tetracycline resistant (MIC ≥ 8 µg/mL). The highest MIC_90_ of tetracycline (32 µg/mL) was noted in bovine and European bison isolates. The most prevalent determinant of tetracycline resistance was *tetW* (in 40.4% of isolates), while *tetA(33)* was detected only in 8.8% of isolates. Four variants of *tetW* (*tetW-1*, *tetW-2*, *tetW-3*, *tetW-4*) were recognized. The *tetW-3* variant was the most frequent and was linked to the ATE-1 transposon. The *tetW-*2 variant, found in a swine isolate, was not previously reported in *T. pyogenes*. This is the first report on determinants of tetracycline resistance in *T. pyogenes* isolates from European bison. These findings highlight that wild animals, including wild ruminants not treated with antimicrobials, can be a reservoir of tetracycline-resistant bacteria carrying resistance determinants, which may be easily spread among pathogenic and environmental microorganisms.

## 1. Introduction

*Trueperella pyogenes*, a Gram-positive irregular rod, is a commensal of the mucus membranes of the upper respiratory, gastrointestinal and urogenital tracts of animals, and as well as an opportunistic pathogen [1,2]. This bacterium can cause different infections, such as mastitis, metritis, pneumonia or abscesses in various organs and tissues in a broad range of livestock, including swine, cattle, goats and sheep [3,4,5,6]. Likewise, *T. pyogenes* purulent infections were reported in dogs and cats [7,8,9]. In addition, infections associated with *T. pyogenes* were also described in various species of wild mammals [10,11,12,13,14] and reptiles [15]. However, diseases caused by *T. pyogenes* are economically important in cattle and swine because they lead to serious losses, including significant losses in milk production and reproduction and a reduction in meat quality [6,16]. Similar effects of *T. pyogenes* infections are also observed in small ruminant breeding [6]. In humans, *T. pyogenes* infections were rarely reported and were mostly associated with occupational exposure through contact with farm animals and their environment [17,18].

Tetracyclines, broad-spectrum antibiotics, are frequently used as the first-choice drugs to prevent and treat human and animal infections, including *T. pyogenes* infections [6]. In addition, in some countries, these antimicrobials are still administrated as growth promoters in animal farming, especially poultry, cattle and swine [19,20]. Tetracycline, oxytetracycline, chlortetracycline and doxycycline are tetracyclines commonly applied in veterinary medicine [21,22]. Oxytetracycline is one of the antimicrobials most often used to treat clinical metritis. However, therapy with the long-acting oxytetracycline is not always a good choice for treatment metritis associated with *T. pyogenes* [23]. Currently, the wide use of tetracyclines is considered to be the main reason for increased antimicrobial resistance among Gram-negative and Gram-positive bacteria [24]. Importantly, the cross-resistance between different tetracyclines is noted. The resistance to tetracyclines is determined by several mechanisms that are supported by tetracycline resistance proteins known as Tet proteins. The most common tetracycline resistance mechanisms include an active efflux of drugs from the bacterial cell, ribosomal protection from drug action, and enzymatic inactivation of drugs [21,24,25]. Until now, 61 different tetracyclines resistance genes (*tet*) encoding Tet proteins, often associated with transposons or plasmids, have been characterized [24]. Due to the relation of the *tet* genes with mobile genetic elements, their distribution among strains, also belonging to different bacterial species, may be strongly widespread.

The tetracycline resistance in *T. pyogenes* was reported in several phenotypic studies, which referred mainly to isolates from cattle and swine [3,5,6,8,16,26,27,28,29,30,31,32,33,34,35]. In the case of isolates from wild animals, only limited data on the tetracycline resistance are available [5,10]. Moreover, genotypes of tetracycline resistance in *T. pyogenes* isolates of various origins are still poorly understood. Till now, two mechanisms of tetracycline resistance in *T. pyogenes* have been described, first associated with ribosomal protection proteins (RPPs) encoded by the *tetW* or *tetM* genes, and second relied on the activity of efflux pump proteins encoded by the *tetK*, *tetL* or *tetA(33)* genes [33,36,37]. Importantly, different antimicrobial resistance genes in *T. pyogenes*, including tetracycline resistance genes, may be associated with transposons [37], plasmids [36] or integron gene cassettes [26,38].

Currently, there are no *T. pyogenes*–specific breakpoints for antimicrobial susceptibility testing available in the Clinical and Laboratory Standards Institute (CLSI) guidelines [39]. Hence, the resistance to antimicrobials commonly used against *T. pyogenes* infections, including tetracyclines, should be incessantly monitored, as obtained data would be important to define missing breakpoints for this bacterium. Thus, in this study, we investigated the prevalence of tetracycline resistance and the distribution and characterization of tetracycline resistance determinants among a large collection of *T. pyogenes,* isolates from different host species, including unique isolates from European bison (*Bison bonasus*).

## 2. Results

### 2.1. Susceptibility to Tetracycline

Among 114 tested *T. pyogenes* isolates from a different origin, 49 (43.0%; CI 95%: 34.3%, 52.2%) were classified as resistant to tetracycline (MIC ≥ 8 µg/mL), and the MIC_50_ and MIC_90_ values for all isolates were 4 and 32 µg/mL, respectively. The distribution of tetracycline MIC values obtained for the studied isolates is presented in Table 1. The highest prevalence of tetracycline resistance was noted among the bovine isolates– 89.5% (34/38; CI 95%: 75.9%, 95.8%). The MIC_50_ and MIC_90_ of tetracycline for bovine isolates had the same value, 32 µg/mL. The significantly lower prevalence of tetracycline resistance was found in isolates from swine–33.3% (9/27; CI 95%: 18.6%, 52.2%; *p* < 0.001), European bison–16.7% (5/30; CI 95%: 7.3%, 33.6%; *p* <0.001) and small ruminants–5.3% (1/19; CI 95%: 0.9%, 24.6%; *p* < 0.001). There was no significant difference in the prevalence of tetracycline resistance between swine and European bison isolates (*p* = 0.219), nor between European bison and small ruminant isolates (*p* = 0.384). However, the prevalence of tetracycline resistance was significantly lower in small ruminants than in swine isolates (p = 0.031). The tetracycline MIC_50_ and MIC_90_ values for swine isolates were 4 and 8 µg/mL, respectively. The MIC_50_ for caprine and European bison isolates was the same, 0.25 µg/mL. However, the MIC_90_ value was different, 1 µg/mL for caprine isolates and 32 µg/mL for European bison isolates. Among small ruminant, *T. pyogenes* isolates, only one originated from a goat was resistant to tetracycline, while all ovine isolates were susceptible to the tested antibiotic, and the MIC_50_ and MIC_90_ values were ≤ 0.125 µg/mL.

### 2.2. Prevalence of Tetracycline Resistance Genes

The prevalence of selected tetracycline resistance genes was investigated for all tested *T. pyogenes* isolates (*n* = 114). The tetracycline resistance genotypes of *T. pyogenes* isolates are phenotypically classified as resistant (49/114), and the occurrence of resistance determinants among these isolates are summarized in Table 2. In 46 isolates out of 49 tetracycline-resistant isolates, the results of PCR with universal primer set indicated the presence of tetracycline resistance genes encoding RPPs. The RPPs genes were found in 32 bovines, nine swine and five European bison isolates. Then the presence of *tetW*, one of the more frequent genes encoding the tetracycline resistance RPPs, was studied by PCR using two different specific primer sets. In PCR with primers tetW_F and tetW_R, previously described [37], the positive result was obtained only for 38 isolates. In the case of 8 remaining isolates, amplicons obtained with universal primers were subjected to sequence analysis. Based on the analysis results, a new primer set for *tetW* was designed. The use of tetW-all_F and tetW-all_R primers (designed in this study) allowed to detection of *tetW* in all 46 *T. pyogenes* isolates, recognized previously as RPPs gene-positive with the universal primer set. Finally, it was confirmed that 46/114 isolates (40.4%; CI 95%: 31.8%, 49.5%) carried the *tetW* gene, including 32 of 38 bovine isolates (84.2%; CI 95%: 69.6%, 92.6%), nine of 27 swine isolates (33.3%; CI 95%: 18.6%, 52.2%) and five of 30 European bison isolates (16.7%; CI 95%: 7.3%, 33.6%).

Among all *T. pyogenes* isolates 8.8% (10/114; CI 95%: 4.8%, 15.4%) carried the *tetA(33)* gene, including nine of 38 bovine isolates (23.7%; CI 95%: 13.0%, 39.2%) and one of 13 caprine isolate (7.7%; CI 95%: 1.4%, 33.3%). The prevalence of the *tetA(33)* gene did not differ significantly between the bovine and caprine isolates (*p* = 0.419). Although, eight of the bovine isolates harbored both resistance genes, *tetA(33)* and *tetW*. Other tested genes, *tetM*, *tetO*, *tetK*, and *tetL*, were not detected in the studied isolates.

Generally, genetic tetracycline resistance determinants were found in 48 out of 49 tetracycline-resistant isolates (98.0%; CI 95%: 89.3%, 99.6%) that indicates the high accordance between the tetracycline resistance phenotype and genotype in the studied isolates (Table 2). Only one tetracycline-resistant bovine isolate (16/B), for which MIC was 8 µg/mL, did not carry any of the tested *tet* genes.

### 2.3. Sequence and Phylogenetic Analysis of the tet Genes

The sequence analysis of PCR products obtained using the universal primer set for tetracycline resistance RPPs genes was performed for 15 isolates, including 8 isolates in which *tetW* was not detected by PCR with the *tetW* primer set previously described [37], and 7 selected *tetW*-positive isolates confirmed by this reaction, used as controls of PCR specificity. The analysis showed that all amplicons should be identified as the *tetW* gene. According to the BLASTN analysis, the *tetW* sequences of six *T. pyogenes* swine isolates (10/S, 11/S, 12/S, 14/S, 17/S, 49/S) displayed 100% identity to each other, as well as 99.89% identity to the *tetW-1* gene of *Butyrivibrio fibrisolvens* (AJ427421.2). However, the *tetW* nucleotide sequence of the 2/S isolate indicated 99.89% identity to *tetW-2* of *Megaspharea elsdenii* (AY485124.1). A group of isolates, including two bovines (2/B, 26/B), one swine (8/S) and four isolates from European bison (3/Z, 8/Z, 10/Z, 14/Z), contained *tetW* displaying 100% identity to the sequence of the *tetW-3* gene related to transposon ATE-1 from *T. pyogenes* (AY049983.2). However, the *tetW* nucleotide sequence of the 16/S isolate showed 100% identity to the *tetW-4* gene associated with transposon ATE-2 from *T. pyogenes* (DQ517519.1).

**Figure 1:**
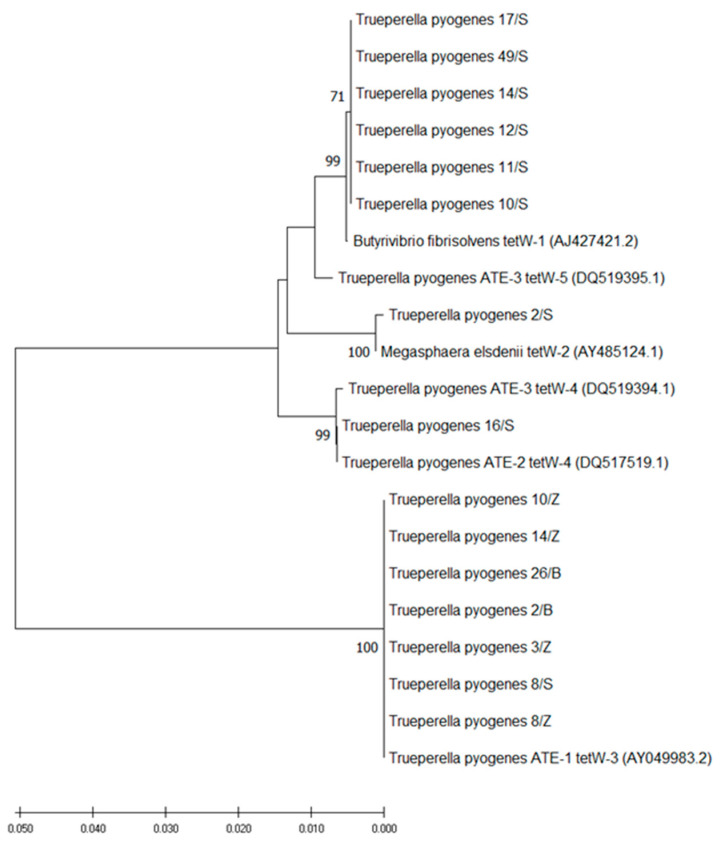
The phylogenetic tree based on a comparison of sequences (933 nucleotides) of the *tetW* gene of 15 *Trueperella pyogenes* isolates from this study (2/B, 26/B, 3/Z, 8/Z, 10/Z, 14/Z, 2/S, 8/S, 10/S, 11/S, 12/S, 14/S, 16/S, 17/S, 49/S) and the *tetW* sequences of selected bacterial species retrieved from the GenBank database (accession numbers in parentheses). The tree was constructed by the neighbor-joining method. Bootstrap values (1000 replicates) of above 70% are shown. The scale bar represents the number of substitutions per site.

The phylogenetic analysis showed that the sequences of *tetW* differed in the studied *T. pyogenes* isolates (Figure 1). Thus, based on noticed diversity, those isolates may be divided into four groups carrying variable variants of the *tetW* gene, such as *tetW-1*, *tetW-2*, *tetW-3* and *tetW-4* (Figure 1). The swine *T. pyogenes* isolates carried four different variants of *tetW*, while bovine and European bison isolates possessed only *tetW-3* (Figure 1).

Importantly, the nucleotide sequences differed among the reported *tetW* variants in the studied isolates (Figure S1 of Supplementary Materials). Sequence analysis revealed that the *tetW* genes from European bison *T. pyogenes* isolates (8/Z and 14/Z) were 100% identity to each other and to the *tetW* sequences previously described in this bacterium (NG_048284.1, AY049983.2). Moreover, *tetW* from these *T. pyogenes* isolates shared 93.84% identity with *tetW* from *Lawsonia intracellularis* (NG_055990.1).

The nucleotide analysis of the *tetA(33)* sequences from two selected bovine *T. pyogenes* isolates (2/B and 26/B) revealed 100% identity with the *tetA(33)* from *T. pyogenes* (AY255627.1) and *Corynebacterium glutamicum* (NG_048127.1). In addition, these genes shared 99.59% identity with *tetA(33)* from *Arthrobacter protophormiae* (DQ077487.1).

### 2.4. Occurrence of tetW-3 Linked to the ATE-1 Transposon Among T. pyogenes Isolates

Based on the results of sequence analysis, *tetW-*3, a variant of the *tetW* gene, was suspected to be the most prevalent tetracycline resistance determinant among studied *T. pyogenes* isolates. To confirm this observation, a presence of the 522 bp fragment (*tetW-3– orf110*) characteristic for the *tetW-3* variant linked to the ATE-1 transposon was investigated. The *tetW-3 or f110* fragment was detected in 38 out of 46 tetracycline-resistant *T. pyogenes* isolates harboring *tetW*. All *tetW*-positive bovine (*n* = 32) and European bison (*n* = 5) isolates carried *tetW-3* linked to ATE-1 transposon (Table 2). However, these genetic elements were found only in one swine isolate out of nine carrying *tetW*. These findings indicate that *tetW-3*, more important, associated with a mobile element, is the predominant tetracycline resistance determinant in *T. pyogenes* isolates from ruminants.

## 3. Discussion

In recent years, increasing antimicrobial resistance in bacteria of animal origin has become an important health and economic issue [40]. One of the well-recognized factors involved in the development of bacterial resistance is the overuse of antimicrobials in veterinary medicine [41]. The use of tetracyclines in food-producing animals in Europe is invariably higher compared to other antimicrobial classes [41]. According to the European Medicines Agency (EMA) tenth European Surveillance of Veterinary Antimicrobial Consumption (ESVAC) report, the sales of tetracyclines for food-producing animals in 2018 was the highest in Cyprus (155.2 mg/PCU) and the lowest in Norway (0.1 mg/PCU), while in Poland it was 47.3 mg/PCU [41]. In Poland, tetracyclines are widely used in livestock, especially in cattle and swine [42]. However, recently, an increase in tetracycline consumption in horses has been observed as well [42]. Moreover, these antimicrobials can also be found in medicinal feeds used for animals [22]. It should be highlighted that widespread use of tetracyclines in animals may lead to significant dissemination of bacteria resistant to these antimicrobials and to environmental accumulation of resistance determinants [43,44]. This problem also concerns the treatment of infections caused by *T. pyogenes* in livestock, mainly in cattle, for which tetracyclines are often used. Thus, in the present study, we investigated the tetracycline resistance mechanisms among *T. pyogenes* isolates of different origins, concerning unique isolates from European bison. Importantly, resistance genotypes and phenotypes were compared to obtain data important for further research on establishing tetracycline breakpoints specific for *T. pyogenes*.

Discussing the results of antimicrobial susceptibility testing should consider methodological differences may cause some interpretation inconsistencies. In the case of many studies, *T. pyogenes* isolates for which a tetracycline MIC was 8 µg/mL or higher were classified as resistant, like in our work [10,26,27,29,33].

In the present study, the high prevalence of tetracycline resistance (89.5%) in bovine *T. pyogenes* isolates (MIC_90_ = 32 µg/mL) was reported. A similar observation was noted by Zastempowska, and Lassa [28] for *T. pyogenes* isolates from bovine mastitis, also collected in Poland, among which 85.5% were reported as resistant to tetracycline. In Iran, the tetracycline resistance of *T. pyogenes* isolated from bovine mastitis and metritis ranges from 10.8% to 97.8% [32,34,35]. Similarly, in China, 70.0% of *T. pyogenes* isolates from bovine mastitis were tetracycline-resistant [31]. In this country, the high-frequency of resistance to tetracycline (68.8%), oxytetracycline (53.1%) and doxycycline (62.5%) was also shown in *T. pyogenes* isolates from bovine endometritis [26,33]. The high percentage, over the range from 41.7% to 54.2%, of tetracycline, chlortetracycline and oxytetracycline-resistant *T. pyogenes* isolates of bovine origin was noted in the United States [8,27]. Moreover, Ozturk et al. [30] reported 84.1% of bovine *T. pyogenes* strains isolated in Turkey as resistant to oxytetracycline. However, the MIC_90_ values of tetracycline, chlortetracycline, oxytetracycline, doxycycline and metacycline determined for *T. pyogenes* isolates from bovine endometritis in China, were 32 µg/mL, 16 µg/mL, 32 µg/mL, 16 µg/mL, and 8 µg/mL, respectively [33]. In Europe, the highest tetracycline MIC_90_ (64 µg/mL) was reported for bovine *T. pyogenes* isolates in Spain and Germany [6,45].

The tetracycline resistance at a relatively lower level has been noted for swine *T. pyogenes* isolates. In this study, 33.3% of swine isolates were classified as resistant to tetracycline, and MIC_90_ was 8 µg/mL. A similar rate of tetracycline-resistant swine *T. pyogenes* isolates, 41.7%, was found in the United States [8]. Furthermore, MIC_90_ determined for swine *T. pyogenes* isolated in Spain was 16 µg/mL [16]. Although, in some cases, higher tetracycline MIC_50_ values were reported for swine *T. pyogenes* isolates comparing to bovine ones, e.g., in the study of Yoshimura et al. [3], chlortetracycline MIC_50_ was 12.5 µg/mL for swine isolates and 6.25 µg/mL for bovine isolates. Differences in the consumption of tetracyclines used for the treatment of infections in swine and cattle seem to be one of the possible reasons for the observed divergence in a level of tetracycline resistance [8].

The resistance to tetracyclines in *T. pyogenes* isolated from small ruminants has been poorly examined to date. In the present study, we noted a low percentage of tetracycline-resistant caprine *T. pyogenes* isolates (7.7%), whereas all isolates from sheep were tetracycline-susceptible. These results confirmed the observations of Galán-Relaño et al. [6] that showed a relatively low prevalence of tetracycline-resistant *T. pyogenes* strains isolated from small ruminants in Spain. However, they demonstrated significantly higher tetracycline MIC_90_ values (16 µg/mL for caprine isolates and 8 µg/mL for ovine isolates) than that reported in our study (1 µg/mL for caprine isolates and ≤ 0.125 µg/mL for ovine isolates) [6]. Moreover, Fernández et al. [46] also reported the high MICs of tetracycline (16 µg/mL) for all tested *T. pyogenes* isolates from sheep in Spain.

It might seem that wild animals living in the environment with no antibiotic pressure are not a reservoir of antimicrobial-resistant bacteria. However, in our study, we demonstrated that wild ruminants, such as European bison, might be infected with tetracycline-resistant *T. pyogenes* strains. Admittedly, the rate of tetracycline-resistant isolates from those wild animals was relatively low (16.7%) but concurrently higher than that for isolates from goats or sheep. Interestingly, MIC_90_ of tetracycline for European bison isolates was the same as obtained for bovine isolates, although MIC_50_ was higher for bovine origin isolates. It should be highlighted that *T. pyogenes* isolates were collected from European bison never treated with any antimicrobials, thus in this case, an effect of selective pressure could be excluded. Our observations suggest a possibility of infection of those wild ruminants by *T. pyogenes* strains of bovine origin. The fact that European bison may use the agricultural land and the same grassland as cattle strongly indicates that transmission of resistant bacteria may occur among livestock and wild animals [47,48]. On the other hand, the fact that tetracycline resistance determinants can be acquired by *T. pyogenes* from other bacteria should also be considered. Similar observations concerning the tetracycline resistance in *T. pyogenes* that occurred in wild herbivores were previously noted for isolates from farmed white-tailed deer (*Odocoileus virginianus*) [10,49]. The occurrence of *T. pyogenes* isolates from cases of pneumonia in this animal species, resistant to chlortetracycline (48.3%) and oxytetracycline (31.0%), was reported. Moreover, MIC_90_ values of these antimicrobials were relatively high, 8 µg/mL and 16 µg/mL for chlortetracycline and oxytetracycline, respectively [10]. Conversely, the lower MIC_90_ (0.19 µg/mL) of tetracycline was reported for *T. pyogenes* isolates from cases of necrobacillosis in white-tailed deer [49].

Although the *T. pyogenes* resistance to tetracyclines has been widely reported, its genetic determinants were not well described. Our study showed that the tetracycline resistance in *T. pyogenes* was mainly associated with the presence of the *tetW* gene. This gene encodes TetW, one of the ribosomal protection proteins associated with tetracycline resistance [50]. The *tetW* gene was previously identified in many microorganisms, among others in anaerobic bacteria isolated from bovine and sheep rumen, swine feces and human fecal biota [51,52,53,54,55]. In the present study, *tetW* was detected in 40.4% of *T. pyogenes* isolates, mainly swine and bovine, classified as resistant, for which the tetracycline MIC values ranged from 8 to 32 µg/mL. Interestingly, Zastempowska and Lassa [28] found the *tetW* gene in all studied bovine *T. pyogenes* isolates from mastitis with the tetracycline MICs greater or equal 4 µg/mL. Generally, the presence of *tetW* in tetracycline-susceptible bacteria has been rarely reported [55]. In the study of Billington et al. [56], the *tetW* gene was the most prevalent in bovine, swine, and macaw *T. pyogenes* isolates resistant to tetracycline, chlortetracycline and oxytetracycline. The presence of this gene among bovine *T. pyogenes* isolates resistant to different tetracyclines was also reported in other studies [33,34,35]. Thus, the TetW protein probably determines resistance to various antimicrobials belonging to the class of tetracyclines. Until now, the data on the occurrence of the *tetW* gene among *T. pyogenes* isolates from wild animals were limited. Only in the case of three *T. pyogenes* isolates from gray slender lorises kept at Zoo, the presence of *tetW* was reported [14]. Moreover, the presence of this gene was also reported in one tetracycline-resistant *T. pyogenes* to isolate from birds [56]. Surprisingly, in our study, a significant percentage of the European bison *T. pyogenes* isolates carried the *tetW* gene as the main tetracycline resistance determinant. To the best of our knowledge, this is the first report on the prevalence of this gene in *T. pyogenes* isolates from European bison.

The differentiation of the *tetW* gene sequences was observed in various bacterial species, such as *Megasphera elsdenii* [53] or *Bifidobacterium* spp. [55,57]. Although, a detailed analysis of the *tetW* sequence has been rarely performed, and the variants of this gene the most often were not determined. Billington and Jost [37] described different sequence variants of the *tetW* gene, including *tetW-1*, *tetW-3*, *tetW-4* and *tetW-5*, found in *T. pyogenes*. Similarly, in the studied *T. pyogenes* isolates of various origin, we detected *tetW-1*, *tetW-3* and *tetW-4* variants of *tetW*. Importantly, in one swine isolate, we found the *tetW-2* variant, which was not previously reported in *T. pyogenes*. In this study, the *tetW-1* and *tetW-4* variants were related only to the swine isolates. However, *tetW-3* was found in swine, bovine and European bison isolates. In contrast, Billington and Jost [37] identified this variant of *tetW* only in bovine isolates. Thus, this is the first description of the *tetW-3* variant occurring in swine and European bison *T. pyogenes* isolates.

Importantly, it was showed that the flanking regions of *tetW* might have different sequences [54,55]. Therefore, a multiple nucleotide sequence alignment analysis was done for selected variants of the *tetW* gene, and the results are presented in Figure S1 of Supplementary Materials. As we suspected, some differences in the sequences of flanking regions of *tetW-1* and *tetW-3* were detected. This finding may explain false-negative results obtained for several isolates by PCR using the previously described primer set [37]. Since the reverse primer (tetW_R) sequence corresponds to the variable flanking region of *tetW*, this gene may not be detected in some tetracycline-resistant *T. pyogenes* isolates. The same problem was noted by Villedieu et al. [57] in a case of some tetracycline-resistant isolates of oral bacteria tested for the *tetW* presence by PCR using described primers. Therefore, in this study, we designed new primers specific for *tetW,* which link to the sequences inside this gene. The proposed primer set allows for the successful detection of all analyzed variants of the *tetW* gene. Nevertheless, all potential differences between sequences of *tetW* variants, especially of flanking regions, should be considered during the phylogenetic analysis of *tetW* relationships. Generally, our observations suggest that the studied *T. pyogenes* isolates might probably acquire the *tetW* gene from different bacterial species.

It is known that the *tetW* gene in *T. pyogenes* may be carried by different transposons, ATE-1, ATE-2 or ATE-3, usually depending on a variant of *tetW* [37]. In this study, the ATE-1 transposon was the most prevalent mobile genetic element related to the *tetW* gene in *T. pyogenes*. The *tetW-3* variant linked to ATE-1 was found mainly in bovine and European bison isolates and in one swine isolate. In a single swine isolate, we found another transposon, ATE-2, carrying *tetW-4,* which was not previously noted in swine *T. pyogenes*. It is the first report on the occurrence of ATE-1 and ATE-2 transposons in *T. pyogenes* of swine origin. The remaining swine isolates in this study contained *tetW-1,* which is not connected to any known transposons in *T. pyogenes*. Interestingly, the *tetW-3* gene found in European bison *T. pyogenes* isolates were linked to ATE-1 like in the bovine isolates. This observation also indicates the potential relationship between *T. pyogenes* isolates from cattle and European bison. Moreover, it seems that ATE-1 is a crucial genetic element involved in the widespread distribution of tetracycline resistance determinants among *T. pyogenes* strains that occurred in ruminants. However, the ATE-3 transposon was not detected in our study. The absence of ATE-3, which is frequently associated with the streptomycin resistance *aadE* gene [37], was not surprising as this gene was not found in the collection of *T. pyogenes* isolates in our previous investigation [38].

In the present study, we also tested the presence of two genes, *tetM* and *tetO*, encoding tetracycline resistance RPPs, TetM and TetO, respectively. Nevertheless, any of those genes were not found in the tested *T. pyogenes* isolates. On the contrary, *tetM* was detected in *T. pyogenes* isolates from bovine endometritis by Zhang et al. [33], while the absence of *tetO* was consistent with our results.

Another tetracycline resistance determinant revealed in the studied *T. pyogenes* isolates was *tetA(33)*. This gene encodes the tetracycline-specific efflux pump protein–TetA(33), a member of the major facilitator superfamily (MFS) of efflux pumps [36]. TetA(33) was previously described in *Corynebacterium glutamicum* as one of two repressor-regulated tetracycline resistance determinants of efflux systems in Gram-positive bacteria [58]. The presence of the *tetA(33)* gene was demonstrated in *T. pyogenes* for the first time by Jost et al. [36]. Additionally, this gene can be found in some whole-genome sequences of *T. pyogenes* deposited in the GenBank database (CP033904.1, CP029004.1, CP029001.1). Our results indicated the relatively low prevalence (8.8%) of the *tetA(33)* gene among the studied *T. pyogenes* isolates. This gene was detected in tetracycline-resistant, mainly bovine and single caprine isolates. To the best of our knowledge, this gene was not previously reported in *T. pyogenes* isolated from goat. It is worth noting that in the case of two isolates, in which *tetA(33)* was the only gene related to the tetracycline resistance phenotype, the MIC of tetracycline was 8 µg/mL. However, for *T. pyogenes* isolates harboring two tetracycline resistance genes, *tetW* and *tetA(33)*, the MIC of tetracycline ranged from 16 to 32 µg/mL. The *tetA(33)* gene in *C. glutamicum* and *T. pyogenes* is associated with the insertion sequence *IS6100* located in a plasmid, pTET3 or pAP2, respectively [36,58]. Additionally, the pAP2 plasmid of *T. pyogenes* may also contain, except *tetA(33)*, a macrolide resistance determinant–*ermX*, whereas a presence of both those genes is not closely related [36]. Surprisingly, *tetA(33)* associated with *IS6100* located in the *T. pyogenes* chromosomal DNA was also reported [59].

Moreover, in our study, we investigated the presence of two other genes, *tetK* and *tetL*, encoding proteins associated with the efflux pump mechanism in *T. pyogenes*. The presence of both these genes in bovine *T. pyogenes* isolates was previously reported by Zhang et al. [33], but they were not detected in this study.

There are still limited data concerning antimicrobials’ destiny in the environment and their effect on the development and emergence of antimicrobial resistance in bacteria. It is well-known that the overuse of antimicrobials in agriculture may lead to an increased selection of resistant strains [60]. However, it seems that one of the essential reasons for a high prevalence of antimicrobial resistance genes in the environment may be the horizontal transfer of these genes from fecal microbiota of livestock to environmental bacteria [61,62]. The *tetW* gene, the most widespread tetracycline resistance determinant in bacteria of different origins, may be a good example of disseminating resistance genes among clinical and environmental strains. The occurrence of *tetW* in soil and water samples nearby of swine and cattle farms is evidence of the persistence of resistance genes in the various environment, including wildlife [52,61,63,64,65]. Our findings obtained for *T. pyogenes* isolated from European bison also confirmed the easy spread of *tetW* among strains that occurred in wild ruminants.

## 4. Materials and Methods

### 4.1. Bacterial Isolates and Culture Conditions

A total of 114 *T. pyogenes* isolates from livestock (38 from cattle, 27 from swine, 13 from goats, six from sheep) and free-living or captive European bison (*n* = 30) in Poland were studied. Clinical specimens were obtained from animals with different types of infections: purulent lesions or abscesses in various tissues (two from cattle, 13 from goats, seven from swine, six from sheep, 12 from European bison), pneumonia (20 from swine, three from European bison), mastitis (26 from cattle), metritis (10 from cattle) and balanoposthitis (15 from European bison). Bacteria were cultured on Columbia Agar supplemented with 5% sheep blood (CAB) (Graso Biotech, Starogard Gdański, Poland) at 37 °C in 5% CO_2_ atmosphere for 48 h. *T. pyogenes* isolates were identified based on the phenotypic properties [5,11]. Additionally, the species-specific pyolysin gene (*plo*) was detected. The sequence of primers and PCR cycling conditions used for *plo* detection are presented in Table 3. The majority of isolates used in this study (*n* = 95) were identified previously [5,11]. The remaining isolates characterized in this investigation (*n* = 19) are described in Table S1 of Supplementary Materials.

Four reference strains, *T. pyogenes* ATCC^®^19411, *T. pyogenes* ATCC^®^49698, *Escherichia coli* ATCC^®^25922 and *Staphylococcus aureus* ATCC^®^25923, were included as controls for antimicrobial susceptibility testing.

### 4.2. Tetracycline Susceptibility Testing

Antimicrobial susceptibility for 95 of the studied isolates was previously carried out by the strip diffusion method using Etest^®^ strips [5]. In this study, tetracycline susceptibility testing for all 114 *T. pyogenes* isolates was performed by the standard microdilution method according to the CLSI guidelines [71]. The bacterial inoculum (approximately 4 × 10^5^ CFU/mL) was prepared in Mueller–Hinton broth (Difco, Franklin Lakes, NJ, USA) containing 5% (*v/v*) fetal calf serum (Graso Biotech, Starogard Gdański, Poland), and 100 µL of the inoculum was added into 96 wells of a microtiter plate. Double serial dilutions of tetracycline (Sigma-Aldrich, Steinheim, Germany) were performed in Mueller–Hinton broth (Difco, Franklin Lakes, New Jersey, USA) containing 5% (*v/v*) fetal calf serum (Graso Biotech, Starogard Gdański, Poland) and then 100 µL of each dilution was added into the respective well, to receive a final tetracycline concentration over the range 128 µg/mL to 0.125 µg/mL. Microtiter plates were incubated at 37 °C in a 5% CO_2_ atmosphere for 24 h. A MIC value was recorded as the lowest concentration of tetracycline that visibly inhibited bacterial growth. In addition, tetracycline concentrations required to inhibit the growth of 50% and 90% of isolates (MIC_50_ and MIC_90_, respectively) were also determined. In the current CLSI guidelines, VET06 and VET08, there are no available tetracycline breakpoints specific for *T. pyogenes* [39,72]. Thus, MIC breakpoints used in this study to classify isolates as susceptible (≤4 µg/mL) or resistant (≥16 µg/mL, also included intermediate, 8 µg/mL) to tetracycline were based on the interpretative criteria recommended for *Corynebacterium* spp. and coryneforms according to the CLSI guidelines [39].

### 4.3. DNA Extraction

A simple boiling method was used for DNA extraction from the tested *T. pyogenes* isolates. Briefly, several colonies from a 48 h culture of an isolate on CAB were suspended in 500 µL of nuclease-free water. The suspension was heated at 99 °C for 10 min, cooled on ice and centrifuged (6 min, 10,500× *g*). The supernatant was collected and stored at -20 °C until further use.

### 4.4. Detection of Tetracycline Resistance Genes

The presence of genes associated with the tetracycline resistance was examined by standard PCR using universal primers for different *tet* genes encoding RPPs, primers specific for both the *tetK* and *tetL* genes, and the primer sets for detecting genes encoding particular tetracycline resistance determinants, such as *tetW*, *tetM*, *tetO*, *tetA(33)*, *tetK,* and *tetL* (Table 3). The *tetW*, *tetM* and *tetO* genes are associated with the ribosomal protection mechanism, while *tetK*, *tetL* and *tetA(33)* with the efflux pump mechanism. Two various pairs of primers were used for *tetW* detection (Table 3). All PCR reactions were performed in a 25 μL reaction mixture containing DreamTaq master mix (2X) (Thermo Fisher Scientific, Waltham, MA, USA), nuclease-free water (Thermo Fisher Scientific, Waltham, MA, USA), 10 pmol of each primer (Genomed, Warsaw, Poland) and 70–90 ng of a template DNA. The thermal cycling conditions are presented in Table 3. Reaction products were recognized by electrophoresis (85 V by 45 min) in 1% (*w/v*) agarose gel in TAE buffer with Midori green DNA stain (Nippon Genetics, Düren, Germany), visualized and analyzed using a VersaDoc Model 1000 imaging system and Quantity One software (version 4.4.0) (Bio-Rad, Hercules, CA, USA). DNA obtained from the clinical isolates, *Enterococcus faecium* TR2 and *Lactobacillus acidophilus* 2499, was used as a positive control in the PCR reactions for *tetM* and *tetK*, respectively. DNA from *T. pyogenes* 2/B and 26/B isolates, after sequencing of PCR products, was applied as a positive control for *tetW* and *tetA(33)* PCR, respectively. The *pVir* plasmid of *Campylobacter jejuni* was a positive control for the *tetO* detection.

### 4.5. Sequencing and Phylogenetic Analysis

The selected amplicons obtained with universal primers for *tet* genes encoding RPPs, as well as with primers specific for the *tetW* and *tetA(33)* genes, were sequenced (Genomed, Warsaw, Poland) in order to confirm the specificity of reactions. In cases of *T. pyogenes* isolates positive in PCR with universal DI_F and DII_R primers, but negative in PCR with primers tetW_F and tetW_R as well with primers specific for other tested genes, the amplicons obtained with the universal primer set were sequenced in order to establish a type of the *tet* gene. All sequencing files were evaluated using the Chromas 2.6.5 software (http://www.technelysium.com.au/chromas.html, accessed on 15 February 2021). Subsequently, the obtained nucleotide sequences were compared with the sequences available in the GenBank database using the nucleotide BLAST program carried out on the National Center for Biotechnology Information (NCBI) website (http://blast.ncbi.nlm.nih.gov, accessed on 15 February 2021) [73]. The alignment was performed using the multiple sequence alignment program Clustal Omega (https://www.ebi.ac.uk/Tools/msa/clustalo/, accessed on 15 February 2021). A phylogenetic analysis was performed for selected isolates based on the sequences of the *tetW* gene obtained by PCR with universal primers. The phylogenetic tree was constructed using the neighbor-joining method [74,75] in MEGA X [76]. The reliability of the tree was evaluated by the bootstrap method with 1000 replications [77].

### 4.6. Detection of tetW-3 Linkage with the ATE-1 T. pyogenes Transposon

A linkage of *tetW-3*, one of the variants of the *tetW* gene, with the ATE-1 transposon was studied by amplification of the 522 bp DNA fragment extending from downstream of *tetW-3* (covered region: 260–281 bp of *tetW-3*) into *orf110* (covered region: 97–118 bp of *orf110*) of the ATE-1 transposon, according to Billington and Jost [37]. The PCR using ATE-1_F and ATE-1_R primers, in conditions presented in Table 3, was performed for all tetracycline-resistant *T. pyogenes* isolates.

### 4.7. Developing of New Primers for tetW Detection

A new primer set was developed to detect the *tetW* gene regardless of its variant. The primers tetW-all_F and tetW-all_R were designed using the Primer-Blast (https://www.ncbi.nlm.nih.gov/tools/primer-blast/, accessed on 15 February 2021) and checked using an Oligo Analysis Tool (https://www.eurofinsgenomics.eu/en/ecom/tools/oligo-analysis/, accessed on 15 February 2021).

### 4.8. Nucleotide Sequence Accession Numbers

The nucleotide sequence of the *tetW* gene from *T. pyogenes* European bison isolate (8/Z) from this study was deposited in GenBank under accession number MT798857. Furthermore, the sequence of the *tetA(33)* gene from the bovine *T. pyogenes* isolate (26/B) was also deposited in GenBank under accession number MT798858.

### 4.9. Statistical Analysis

Categorical variables were presented as a count and frequency in a group and compared between groups using the two-tailed Fisher’s exact test. The Wilson score method was used to calculate 95% confidence intervals (CI 95%) for percentages. A significance level (α) was set at 0.05. Statistical analysis was performed in TIBCO Statistica 13.3.0 (TIBCO Software Inc., Palo Alto, CA, USA).

## 5. Conclusions

The present study provides significant data about the tetracycline resistance mechanisms among *T. pyogenes* isolates from livestock and European bison. Our findings suggest that not only bovine and swine *T. pyogenes* isolates, but also strains prevalent in wildlife may be a source of the tetracycline resistance genes. Moreover, it was confirmed that two main resistance mechanisms: one associated with ribosomal protection proteins encoded by different variants of the *tetW* gene linked to the ATE-1 or ATE-2 transposons, and another related to active efflux pump proteins encoded by the *tetA(33)* gene, determine resistance to tetracycline in *T. pyogenes*. Both mentioned genes may be acquired. However, the *tetW* gene is the most prevalent tetracycline resistance determinant in this bacterium. Most importantly, the presence of *tetW* among *T. pyogenes* isolates from European bison was reported in our study for the first time. Thus, these wild ruminants should be considered as a potential reservoir of tetracycline-resistant *T. pyogenes* strains. Nevertheless, further investigation on determinants of tetracycline resistance and their association with mobile genetic elements in *T. pyogenes* are needed, especially to improve interpretive criteria important for susceptibility testing, and consequently to use the most appropriate antibiotic treatment of infections caused by this pathogen.

## Figures and Tables

**Table 1:** Distribution of minimum inhibitory concentration (MIC) of tetracycline, MIC_50_ and MIC_90_ values for the studied *Trueperella pyogenes* isolates (*n* = 114).

Isolate Origin	Number of Isolates with the Indicated MIC (µg/mL)^a^											MIC_50_	MIC_90_
	≤ 0.125	0.25	0.5	1	2	4	8	16	32	64	≥128		
Cattle	1	1	2				4	9	21			32	32
Swine	1	3	1	1	1	11	6	2	1			4	8
Goat	4	5	2	1			1					0.25	1
Sheep	5		1									≤0.125	≤0.125
European bison	11	9	4		1			1	4			0.25	32
**Total**	22	18	10	2	2	11	11	12	26			4	32

^a^ MIC breakpoint for tetracycline used in this study: ≥ 8 µg/mL. Resistant isolates are shaded.

**Table 2:** Occurrence of the *tetW* and *tetA(33)* genes, *tetW-3* linked to ATE-1 transposon, and the tetracycline MIC values for the tetracycline-resistant *Trueperella pyogenes* isolates (*n* = 49). The presence of *tetW* was tested using two different primer sets.

Isolate Designation	Isolate Origin	Gene^a^				*tetW-3* Linked to ATE-1 ^e^	MIC (µg/mL)
		*Tet* ^b^	*tetW* ^c^	*tetW* ^d^	*tetA(33)*		
2/B	Bovine	+	+	+	+	+	32
4/B	Bovine	+	+	+	+	+	32
5/B	Bovine	+	+	+	-	+	32
6/B	Bovine	+	+	+	-	+	16
7/B	Bovine	+	+	+	+	+	32
8/B	Bovine	+	+	+	-	+	32
9/B	Bovine	+	+	+	-	+	16
10/B	Bovine	+	+	+	-	+	32
11/B	Bovine	+	+	+	-	+	16
12/B	Bovine	+	+	+	-	+	32
14/B	Bovine	+	+	+	-	+	16
15/B	Bovine	-	-	-	+	-	8
16/B	Bovine	-	-	-	-	-	8
18/B	Bovine	+	+	+	-	+	32
19/B	Bovine	+	+	+	-	+	32
20/B	Bovine	+	+	+	-	+	32
21/B	Bovine	+	+	+	+	+	32
22/B	Bovine	+	+	+	-	+	32
23/B	Bovine	+	+	+	+	+	32
24/B	Bovine	+	+	+	-	+	32
25/B	Bovine	+	+	+	-	+	32
26/B	Bovine	+	+	+	+	+	16
27/B	Bovine	+	+	+	-	+	32
28/B	Bovine	+	+	+	-	+	32
29/B	Bovine	+	+	+	-	+	16
30/B	Bovine	+	+	+	-	+	16
31/B	Bovine	+	+	+	-	+	16
32/B	Bovine	+	+	+	+	+	32
33/B	Bovine	+	+	+	+	+	32
34/B	Bovine	+	+	+	-	+	8
35/B	Bovine	+	+	+	-	+	32
36/B	Bovine	+	+	+	-	+	16
37/B	Bovine	+	+	+	-	+	8
38/B	Bovine	+	+	+	-	+	32
2/S	Swine	+	-	+	-	-	16
8/S	Swine	+	+	+	-	+	32
10/S	Swine	+	-	+	-	-	8
11/S	Swine	+	-	+	-	-	8
12/S	Swine	+	-	+	-	-	8
14/S	Swine	+	-	+	-	-	8
16/S	Swine	+	-	+	-	-	16
17/S	Swine	+	-	+	-	-	8
49/S	Swine	+	-	+	-	-	8
3/Z	European bison	+	+	+	-	+	32
7/Z	European bison	+	+	+	-	+	32
8/Z	European bison	+	+	+	-	+	16
10/Z	European bison	+	+	+	-	+	32
14/Z	European bison	+	+	+	-	+	32
6/K	Caprine	-	-	-	+	-	8

^a^ +: presence of a gene; -: absence of a gene; ^b^ gene detected using universal primers detecting tetracycline resistance genes encoding ribosome protection proteins; ^c^ gene detected using primers designed by Billington and Jost [37]; ^d^ gene detected using new primers designated in this study; ^e^ presence of 522 bp fragment indicating the presence of *tetW-3* linked to ATE-1 transposon.

**Table 3:** Primers and PCR conditions used in this study.

Primer Designation	Primer Sequence (5’–3’)	Target Gene	Annealing Temperature (°C)	Amplicon Size (bp)	Reference
plo_F plo_R	TCATCAACAATCCCACGAAGAG TTGCCTCCAGTTGACGCTTT	*plo*	60 ^b^	150	[27]
DI_F DII_R	GAYACICCIGGICAYRTIGAYTT GCCCARWAIGGRTTIGGIGGIACYTC	*tet^a^*	53 ^b^	1100	[66]
TKI_F TL32_R	CCTGTTCCCTCTGATAAA CAAACTGGGTGAACACAG	*tetK/* *tetL*	50 ^b^	1050	[67]
tetW_F tetW_R	GACAACGAGAACGGACACTATG CGCAATAGCCAGCAATGAACGC	*tetW*	58 ^b^	1843	[37]
tetM_F tetM_R	TTAAATAGTGTTCTTGGAG CTAAGATATGGCTCTAACAA	*tetM*	54 ^c^	656	[68]
tetA(33)_F tetA(33)_R	GATGCCGATTCTTCCCGCACTGC CCACGCATGATGAGAATCACGC	*tetA(33)*	58 ^b^	1089	[36]
tetO_F tetO_R	GGCGTTTTGTTTATGTGCG ATGGACAACCCGACAGAAGC	*tetO*	50 ^c^	559	[69]
tetK_F tetK_R	TATTTGGCTTTGTATTCTTTCAT GCTATACCTGTTCCCTCTGATAA	*tetK*	50 ^b^	1159	[70]
tetL_F tetL_R	ATAAATTGTTTCGGGTCGGAAT AACCAGCCAACTAATGACAATGAT	*tetL*	50 ^b^	1077	[70]
ATE-1_F ATE-1_R	TGCCTGGCAGCGTCCGTCCGTG AGGGCCAAGACCGCCGAGTTCC	*tetW-3–orf110*	55 ^c^	522	[37]
tetW-all_F tetW-all_R	GTCTGTTCGGGATAAGCTCT TGGAATACGCATCTCTGTGA	*tetW*	54 ^c^	466	This study

^a^ Universal primers detecting the tetracycline resistance genes encoding ribosome protection proteins; ^b^ PCR conditions: initial denaturation at 95 °C for 3 min; 35 cycles of denaturation at 95 °C for 1 min, annealing for 1 min at variable temperatures and extension at 72 °C for 2 min; a final extension at 72 °C for 5 min; ^c^ PCR conditions: initial denaturation at 95 °C for 3 min; 30 cycles of denaturation at 95 °C for 45 sec, annealing for 45 sec at variable temperatures and extension at 72 °C for 1 min; a final extension at 72 °C for 2 min.

## Data Availability

The data presented in this study are available in the article or supplementary material.

## Supplementary Materials

The following are available online at https://www.mdpi.com/article/10.3390/antibiotics10040380/s1, Figure S1: Figure S1. Multiple nucleotide sequence alignment of different variants of the tetW gene., Table S1: Table S1. Origin and characteristics of Trueperella pyogenes isolates (n=19) identified in this study, which have not been previously described.
