# Supplementary material for: Trueperella pyogenes Isolates from Livestock and European Bison (Bison bonasus) as a Reservoir of Tetracycline Resistance Determinants
Source: Antibiotics (Basel). 2021 Apr 3;10(4):380. doi: 10.3390/antibiotics10040380 (PMC8065510; doi:10.3390/antibiotics10040380)
Supplement: Supplementary file 1 [file antibiotics-10-00380-s001.zip › antibiotics-1130529-supplementary/Supplementary Materials - Kwiecie¿╜ Ewelina/Figure S1.pdf]

```
tetW-3     ATGAACATTATCAATATCGGGATTCTTGCCCATGTAGATGCGGGCAAGACGACACTGACA    60
tetW-4     ATGAAAATAATCAATATTGGAATTCTTGCCCATGTAGACGCTGGAAAGACGACCTTGACG    60
tetW-5     ATGAAAATAATCAATATTGGAATTCTTGCCCATGTAGACGCTGGAAAGACGACCTTGACG    60
tetW-1     ATGAAAATAATCAATATTGGAATTCTTGCCCATGTAGACGCTGGAAAGACGACCTTGACG    60
tetW-2     ATGAAAATAATCAATATTGGAATTCTTGCCCATGTAGACGCTGGAAAGACGACCTTGACG    60
           ***** ** ******** ** ***************** ** ** ********  **** 

tetW-3     GAAAGCCTGCTGTATGCCAGCGGAACCATTTCAGAGCCGGGGAGCGTCGAAAAAGGGACA    120
tetW-4     GAGAGCCTGCTATATGCCAGCGGAGCCATTTCAGAACCGGGGAGCGTCGAAAAAGGGACA    120
tetW-5     GAGAGCCTGCTATATGCCAGCGGAGCCATTTCAGAACCGGGGAGCGTCGAAAAAGGGACA    120
tetW-1     GAGAGCCTGCTATATGCCAGCGGAGCCATTTCAGAACCGGGGAGCGTCGAAAAAGGGACA    120
tetW-2     GAGAGCCTGCTATATGCCAGCGGAGCCATTTCAGAACCGGGGAGCGTCGAAAAAGGGACA    120
           ** ******** ************ ********** ************************

tetW-3     ACGAGAACGGACACTATGTTTTTGGAGCGGCAGCGTGGAATTACCATTCAAACGGCAGTC    180
tetW-4     ACGAGGACGGACACCATGTTTTTGGAGCGGCAGCGTGGGATTACCATTCAAGCGGCAGTC    180
tetW-5     ACGAGGACGGACACCATGTTTTTGGAGCGGCAGCGTGGGATTACCATTCAAGCGGCAGTC    180
tetW-1     ACGAGGACGGACACCATGTTTTTGGAGCGGCAGCGTGGGATTACCATTCAAGCGGCAGTC    180
tetW-2     ACGAGGACGGACACCATGTTTTTGGAGCGGCAGCGTGGGATTACCATTCAAGCGGCAGTC    180
           ***** ******** *********************** ************ ********

tetW-3     ACTTCTTTCCAGTGGCACAGTTGTAAAGTCAACATTGTGGATACTCCCGGCCACATGGAT    240
tetW-4     ACTTCCTTCCAGTGGCACAGATGTAAAGTCAACATTGTGGATACGCCCGGCCACATGGAT    240
tetW-5     ACTTCCTTCCAGTGGCACAGATGTAAAGTTAACATTGTGGATACGCCCGGCCACATGGAT    240
tetW-1     ACTTCCTTCCAGTGGCACAGATGTAAAGTTAACATTGTGGATACGCCCGGCCACATGGAT    240
tetW-2     ACTTCCTTCCAGTGGCACAGATGTAAAGTCAACATTGTGGATACGCCCGGCCACATGGAT    240
           ***** ************** ******** ************** ***************

tetW-3     TTCTTGGCAGAGGTATACCGCTCTCTGGCCGTTTTGGACGGGGCCATCTTGGTGCTCTCC    300
tetW-4     TTTTTGGCGGAGGTGTACCGCTCTTTGGCTGTTTTAGATGGGGCCATCTTGGTGATCTCC    300
tetW-5     TTTTTGGCGGAGGTGTACCGCTCTTTGGCTGTTTTAGATGGGGCCATCTTGGTGATCTCC    300
tetW-1     TTTTTGGCGGAGGTGTACCGCTCTTTGGCTGTTTTAGATGGGGCCATCTTGGTGATCTCC    300
tetW-2     TTTTTGGCGGAGGTGTACCGCTCTTTGGCTGTTTTAGATGGGGCCATCTTGGTGATCTCC    300
           ** ***** ***** ********* **** ***** ** *************** *****

tetW-3     GCTAGAGATGGCGTACAGGCCCAGACCCGAGTTCTGTTCCATGCCCTACGGAAATTGAAC    360
tetW-4     GCTAAAGATGGCGTGCAGGCCCAGACCCGTATTCTGTTCCATGCCCTGCGGAAAATGAAC    360
tetW-5     GCTAAAGATGGCGTGCAGGCCCAGACCCGTATTCTGTTCCATGCCCTGCGGAAAATGAAC    360
tetW-1     GCTAAAGATGGCGTGCAGGCCCAGACCCGTATTCTGTTCCATGCCCTGCGGAAAATGAAC    360
tetW-2     GCTAAAGATGGCGTGCAGGCCCAGACCCGTATTCTGTTCCATGCCCTGCGGAAAATGAAC    360
           **** ********* **************  **************** ****** *****

tetW-3     ATCCCCACCATTATCTTTATCAACAAGATCGACCAGGTTGACATTGATTTGGAGGGCGTA    420
```

```
tetW-4       ATTCCCACCGTTATCTTTATCAACAAGATCGACCAGGCTGGCGTTGATTTGCAGAGCGTG    420
tetW-5       ATTCCCACCGTTATCTTTATCAACAAGATCGACCAGGCTGGCGTTGATTTGCAGAGCGTG    420
tetW-1       ATTCCCACCGTTATCTTTATCAACAAGATCGACCAGGCTGGCGTTGATTTGCAGAGCGTG    420
tetW-2       ATTCCCACCGTTATCTTTATCAACAAGATCGACCAGGCTGGCGTTGATTTGCAGAGCGTG    420
             ** ****** *************************** ** * ******** ** **** 

tetW-3       TATCAGTCTGTTCGGGATAAGCTCTCCGCCGATATTATCATCAAGCAGACGGTATCGCTG    480
tetW-4       GTTCAGTCTGTTCGGGATAAGCTCTCCGCCGATATTATCATCAAGCAGACGGTGTCGCTG    480
tetW-5       GTTCAGTCTGTTCGGGATAAGCTCTCCGCCGATATTATCATCAAGCAGACGGTGTCGCTG    480
tetW-1       GTTCAGTCTGTTCGGGATAAGCTCTCCGCCGATATTATCATCAAGCAGACGGTGTCGCTG    480
tetW-2       GTTCAGTCTGTTCGGGATAAGCTCTCCGCCGATATTATCATCAAGCAGACGGTGTCGCTG    480
               *************************************************** ******

tetW-3       TCCCCGGAAATAGTTCTGGAGGAAAATACCGACATAGAAGCATGGGATGCGGTCATCGAA    540
tetW-4       TCCCCGGAAATAGTCCTGGAGGAAAATACCGACATAGAAGCATGGGATGCGGTCATCGAA    540
tetW-5       TCCCCGGAAATAGTCCTGGAGGAAAATACCGACATAGAAGCATGGGATGCGGTCATCGAA    540
tetW-1       TCCCCGGAAATAGTCCTGGAGGAAAATACCGACATAGAAGCATGGGATGCGGTCATCGAA    540
tetW-2       TCCCCGGAAATAGTCCTGGAGGAAAATACCGACATAGAAGCATGGGATGCGGTCATCGAA    540
             ************** *********************************************

tetW-3       AATAACGATGGATTATTGGAAAAGTATATCGCAGGAGAGCCAATCAGCCGGGAAGAACTT    600
tetW-4       AATAACGATAAATTATTGGAAAAGTATATCGCAGGAGAACCAATCAGCCGGGAAAAACTT    600
tetW-5       AATAACGATGAATTATTGGAAAAGTATATCGCAGGAGAACCAATCAGCCGGGAAAAACTT    600
tetW-1       AATAACGATGAATTATTGGAAAAGTATATCGCAGGAGAACCAATCAGCCGGGAAAAACTT    600
tetW-2       AATAACGATGCATTATTGGAAAAGTATATCGCAGGAGAACCAATCAGCCAGGAAAAACTT    600
             *********  *************************** ********** **** *****

tetW-3       GCGCGGGAGGAACAGCGGCGGGTTCAAGCCGCTTCCCTGTTCCCAGTCTATCATGGTAGC    660
tetW-4       GTGCGGGAGGAACAGCGGCGGGTTCAAGACGCCTCCCTGTTCCCGGTCTATTATGGCAGC    660
tetW-5       GCGCGGGAGGAACAGCGGCGGGTTCAAGACGCCTCCCTGTTCCCGGTCTATCATGGCAGC    660
tetW-1       GCGCGGGAGGAACAGCAGCGGGTTCAAGACGCCTCCCTGTTCCCAGTCTATCATGGCAGC    660
tetW-2       GCGCGGGAGGAACAGCGGCGGGTTCAAGAAGCCTCCCTGTTTCCGGTCTATCATGGCAGC    660
             * ************** ***********  ** ******** ** ****** **** ***

tetW-3       GCCAAAAACGGCCTTGGCATTCAACGGTTGATGGATGCGGTGATAGGGCTGTTCCAACCG    720
tetW-4       GCCAAAAAGGGCCTTGGCATTCAACCGTTGATGGATGCGGTGACAGGGCTGTTCCAACCG    720
tetW-5       GCCAAAAATGGCCTTGGCATTCAACCGTTGATGGATGCGGTGACAGGGCTGTTCCAACCG    720
tetW-1       GCCAAAAATGGCCTTGGCATTCAACCGTTGATGGATGCGGTGACAGGGCTGTTCCAACCG    720
tetW-2       GCCAAAAAGGGCCTTGGCATTCAACCGTTGATGGATGCGGTGACAGGACTGTTCCAACCG    720
             ******** **************** ***************** *** ************

tetW-3       ACCAAGGAACAGGGGCGCACCGCCCTGTGCGGCAGCGTTTTCAAGGTGGAGTATACAGAT    780
tetW-4       ATTGGGGAACAGGGGAGCGCCGCCCTATGCGGCAGCGTTTTCAAGGTGGAGTATACAGAT    780
```

```
tetW-5      ATTGGGGAACAGGGGGGCGCCGCCCTATGCGGCAGCGTTTTCAAGGTTGAGTACACCGAT    780
tetW-1      ATTGGGGAACAGGGGGGCGCCGCCCTATGCGGCAGCGTTTTCAAGGTTGAGTACACCGAT    780
tetW-2      ATTGGGGAACAGGGGAGCGCCACCCTATGCGGCAGCGTTTTCAAGGTTGAGTACACCGAT    780
            *    ********** ** ** **** ******************** ***** ** ***

tetW-3      TGCGGCCAGAGGCTTGTCTATCTGCGGCTATACAGCGGAACGCTGCGTCTGCGGGATACG    840
tetW-4      TGCGGCCAGCGGCGTGTCTATCTACGGCTATACAGCGGAACGCTGCGCCTGCGGGATACG    840
tetW-5      TGCGGCCAGCGGCGTGTCTATCTACGGTTATACAGCGGAACGCTGCGCCTGCGGGATACG    840
tetW-1      TGCGGCCAGCGGCGTGTCTATCTACGGTTATACAGCGGAACGCTGCGCCTGCGGGATACG    840
tetW-2      TGCGGCCAGCGGCGTGTCTATCTGCGGCTATACAGCGGAACGCTGCGCCTGCGGGATACG    840
            ********* *** ********* *** ******************* ************

tetW-3      GTGGCCCTGGCCGGGAGAGAAAAGCTGAAAATCACAGAGATGCGTATTCCATCCAAAGGG    900
tetW-4      GTGGCCCTGGCCGGGAGAGAAAAGCTGAAAATCACAGAGATGCGTATTCCATCCAAAGGG    900
tetW-5      GTGGCCCTGGCCGGGAGAGAAAAGCTGAAAATCACAGAGATGCGTATTCCATCCAAAGGG    900
tetW-1      GTGGCCCTGGCCGGGAGAGAAAAGCTGAAAATCACAGAGATGCGTATTCCATCCAAAGGG    900
tetW-2      GTGGCCCTGGCCGGGAGAGAAAAGCTGAAAATCACAGAGATGCGTATTCCATCCAAAGGG    900
            ************************************************************

tetW-3      GAGATTGTTCGGACAGATACCGCCCATAAGGGCGAAATTGTCATCCTTCCCAGCGACAGT    960
tetW-4      GAAATTGTTCGGACAGACACCGCTTATCCGGGTGAAATTGTTATCCTTCCCAGCGACAGC    960
tetW-5      GAAATTGTTCGGACAGACACCGCTTATCAGGGTGAAATTGTTATCCTTCCCAGCGACAGC    960
tetW-1      GAAATTGTTCGGACAGACACCGCTTATCAGGGTGAAATTGTTATCCTTCCCAGCGACAGC    960
tetW-2      GAAATTGTTCGGACAGACACCGCTTATCCGGGCGAAATTGTTATCCTTCCCAGCGACAGC    960
            ** ************** *****  **  *** ******** *****************

tetW-3      TTGAGATTAAACGATATATTGGGGGACAAAACCCAACTTCCTCGTGAAATGTGGAGTGAT    1020
tetW-4      GTGAGGTTAAACGATGTATTAGGGGACCCAACCCGGCTCCCTCGTAAAAGGTGGCGTGAG    1020
tetW-5      GTGAGGTTAAACGATGTATTAGGGGACCCAACCCGGCTCCCTCGTAAAAGGTGGCGTGAG    1020
tetW-1      GTGAGGTTAAACGATGTATTAGGGGACCAAACCCGGCTCCCTCGTAAAAGGTGGCGCGAG    1020
tetW-2      GTGAGGTTAAACGATGTATTAGGGGATCAAACCCGGCTCCCTCGTAAAAGGTGGCGCGAG    1020
             **** ********* **** *****   *****  ** ****** *** **** * **

tetW-3      GCTCCCTTCTCTATGCTGCGGACAACGATTACGCCAAAAACGGCAGAGCAAAGAGATCGG    1080
tetW-4      GACCCCCTCCCCATGCTGCGGACGTCGATTGCGCCGAAAACGGCAGCGCAAAGAGAACGG    1080
tetW-5      GACCCCCTCCCCATGCTGCGGACGTCGATTGCGCCGAAAACGGCAGCGCAAAGAGAACGG    1080
tetW-1      GACCCCCTCCCCATGCTGCGGACGACGATTGCGCCGAAAACGGCAGCGCAAAGAGAACGG    1080
tetW-2      GCCCCCCTCCCCATGCTGCGGACGACGATTGCGCCGAAAACGGCAGCGCAAAGAGAACGG    1080
            *  *** ** * ***********  ***** **** ********** ********* ***

tetW-3      TTGCTGGACGCTCTTACGCAAATTGCGGATACTGACCCGCTTTTGTGCTACGAGGTGGAT    1140
tetW-4      CTGCTGGACGCTCTTACGCAACTTGCGGATACTGACCCGCTTTTGCGCTGCGAGGTGGAT    1140
tetW-5      CTGCTGGACGCTCTTACGCAACTTGCGGATACTGACCCGCTTTTGCGCTGCGAGGTGGAT    1140
```

```
tetW-1      CTGCTGGACGCTCTTACGCAACTTGCGGATACTGACCCGCTTTTGCGTTGCGAAGTGGAT    1140
tetW-2      CTGCTGGACGCTCTTACGCAACTTGCGGATACTGACCCGCTTTTGCGCTGCGAAGTGGAT    1140
             ******************** *********************** * * *** ******

tetW-3      TCCATCACCCAAGAGATCATTCTTTCTTTTTTGGGCCGGGTGCAGTTGGAGGTTGTTTCC    1200
tetW-4      TCCATCACCCATGAGATCATTCTTTCTTTTTTGGGCCGGGTGCAGTTGGAGGTTGTTTCC    1200
tetW-5      TCCATCACCCATGAGATCATTCTTTCTTTTTTGGGCCGGGTGCAGTTGGAGGTTGTTTCC    1200
tetW-1      TCCATCACCCATGAGATCATTCTTTCTTTTTTGGGCCGGGTGCAGTTGGAGGTTGTTTCC    1200
tetW-2      TCCATCACCCATGAGATCATTCTTTCTTTTTTGGGCCGGGTGCAGTTGGAGGTCGTTTCC    1200
            *********** ***************************************** ******

tetW-3      GCTTTGCTGGCGGAAAAGTATAAGATTGAAACAGCGGTGAAGGAACCCACCGTCATTTAT    1260
tetW-4      GCTTTGCTGTCGGAAAAATACAAGCTTGAAACAGTGGTAAAGGAACCCACCGTCATTTAT    1260
tetW-5      GCTTTGCTGTCGGAAAAATACAAGCTTGAAACAGTGGTAAAGGAACCCACCGTCATTTAT    1260
tetW-1      GCTTTGCTGTCGGAAAAATACAAGCTTGAAACAGTGGTAAAGGAACCCTCCGTCATTTAT    1260
tetW-2      GCTTTGCTGTCGGAAAAATACAAGATTGAAACAGTGGTAAAGGAACCCACCGTCATTTAT    1260
            ********* ******* ** *** ********* *** ********* ***********

tetW-3      TTAGAGCGGCCGCTCAAAGTGGCCAGCCACACCATCCATATCGAGGTGCCGCCCAACCCG    1320
tetW-4      ATGGAGCGGCCGCTCAAAGCAGCCAGCCACACCATCCATATCGAGGTGCCGCCCAACCCG    1320
tetW-5      ATGGAGCGGCCGCTCAAAGCAGCCAGCCACACCATCCATATCGAGGTGCCGCCCAACCCG    1320
tetW-1      ATGGAGCGGCCGCTCAAAGCAGCCAGCCACACCATCCATATCGAGGTGCCGCCCAACCCG    1320
tetW-2      ATGGAGCGGCCGCTCAAAGCAGCCAGCCACACCATCCATATCGAGGTGCCGCCCAACCCG    1320
             * ****************  ***************************************

tetW-3      TTTTGGGCATCCATCGGACTGTCTGTTACGCCGCTCCCGCTTGGCTCCGGTGTACAATAC    1380
tetW-4      TTTTGGGCATCCATCGGACTGTCTGTTACACCACTCCCGCTTGGCTCCGGTGTACAATAC    1380
tetW-5      TTTTGGGCATCCATCGGACTGTCTGTTACACCACTCCCGCTTGGCTCCGGTGTACAATAC    1380
tetW-1      TTTTGGGCATCCATAGGACTGTCTGTTACACCACTCTCGCTTGGCTCCGGTGTACAATAC    1380
tetW-2      TTTTGGGCATCTATCGGACTGTCTGTTACACCACTCCCGCTTGGCTCCGGCGTACAATAC    1380
            *********** ** ************** ** *** ************* *********

tetW-3      GAGAGCCGGGTTTCCCTGGGATACTTGAACCAGAGTTTTCAAAACGCTGTCATGGATGGT    1440
tetW-4      GAGAGCCGGGTTTCGCTGGGATACTTGAACCAGAGTTTTCAAAACGCTGTCAGGGATGGT    1440
tetW-5      GAGAGCCGGGTTTCGCTGGGATACTTGAACCAGAGTTTTCAAAACGCTGTCAGGGATGGT    1440
tetW-1      GAGAGCCGGGTTTCGCTGGGATACTTGAACCAGAGTTTTCAAAACGCTGTCAGGGATGGT    1440
tetW-2      GAGAGCCGGGTTTCGCTGGGATACTTGAACCAGAGTTTTCAAAACGCTGTCAGGGATGGT    1440
            ************** ************************************* *******

tetW-3      ATCCGTTACGGTCTGGAGCAAGGCTTGTGTGGCTGGAACGTAACGGACTGTAAGATTTGC    1500
tetW-4      ATCCGTTACGGGCTGGAGCAGGGCTTGTTCGGCTGGAACGTAACGGACTGTAAGATTTGC    1500
tetW-5      ATCCGTTACGGGCTGGAGCAGGGCTTGTTCGGCTGGAACGTAACGGACTGTAAGATTTGC    1500
tetW-1      ATCCGTTACGGGCTGGAGCAGGGCTTGTTCGGCTGGAACGTAACGGACTGTAAGATTTGC    1500
```

```
tetW-2       ATCCGTTACGGGCTGGAGCAGGGCTTGTTCGGCTGGAACGTAACGGACTGTAAGATTTGC     1500
             *********** ******** *******  ******************************

tetW-3       TTTGAATACGGGCTTTATTATAGCCCGGTCAGCACGCCGGCGGACTTTCGCTCATTGGCC     1560
tetW-4       TTTGAATACGGGCTTTATTACAGTCCGGTCAGCACGCCGGCGGACTTCCGCTCATTGGCC     1560
tetW-5       TTTGAATACGGGCTTTATTACAGTCCGGTCAGCACGCCGGCGGACTTCCGCTCATTGGCC     1560
tetW-1       TTTGAATACGGGCTTTATTACAGTCCGGTCAGCACGCCGGCGGACTTCCGCTCATTGGCC     1560
tetW-2       TTTGAATACGGGCTTTATTACAGTCCAGTCAGCACGCCGGCGGACTTCCGCTCATTGGCC     1560
             ******************** ** ** ******************** ************

tetW-3       CCGATTGTATTGGAACTGGCATTAAAGGAATCAGGGACACAGTTGCTGGAACCTTATCTC     1620
tetW-4       CCGATTGTATTGGAACAGGCATTGAAGGAATCAGGGACGCAACTGCTGGAACCTTATCTC     1620
tetW-5       CCGATTGTATTGGAACAGGCATTGAAGGAATCGGGGACGCAGCTGCTGGAACCTTATCTC     1620
tetW-1       CCGATTGTATTGGAACAGGCATTGAAGGAATCGGGGACGCAGCTGCTGGAACCTTATCTC     1620
tetW-2       CCGATTGTATTGGAACAGGCATTGAAGGAATCGGGGACGCAGCTGCTGGAACCTTATCTC     1620
             **************** ****** ******** ***** **  *****************

tetW-3       TCCTTCACCCTCTATGCGCCCCAGGAATACCTTTCCAGGGCTTATCATGATGCGCCGAAA     1680
tetW-4       TCCTTCACCCTCTATGCGCCCCGGGAATATCTTTCCAGGGCTTATCATGATGCACCGAAA     1680
tetW-5       TCCTTCACCCTCTATGCGCCCCGGGAATATCTTTCCAGGGCTTATCATGATGCACCGAAA     1680
tetW-1       TCCTTCATCCTCTATGCGCCCCAGGAATACCTTTCCAGGGCTTATCATGATGCACCGAAA     1680
tetW-2       TCCTTCACCCTCTATGCGCCCCAGGAATACCTTTCCAGGGCTTATCATGATGCACCGAAA     1680
             ******* ************** ****** *********************** ******

tetW-3       TATTGTGCCACCATCGAAACGGCCCAGATAAAAAAGGATGAAGTTGTCTTTACTGGCGAG     1740
tetW-4       TACTGTGCCACCATCGAAACGGTCCAGGTAAAAAAGGATGAAGTTGTCTTTACTGGCGAG     1740
tetW-5       TACTGTGCCACCATCGAAACGGTCCAGGTAAAAAAGGATGAAGTTGTCTTTACTGGCGAG     1740
tetW-1       TACTGTGCCACCATCGAAACGGCCCAGGTAAAAAAGGATGAAGTTGTTTTTACTGGCGAG     1740
tetW-2       TACTGTGCCACCATCGAAACGGCCCAGGTAAAAAAGGATGAAGTTGTCTTTACTGGCGAG     1740
             ** ******************* **** ******************* ************

tetW-3       ATTCCCGCCCGTTGCATACAGGCATACCGTACTGATTTGGCCTCTTACACCAATGGGCGG     1800
tetW-4       ATTCCCGCCCGCTGTATACAGGCATACCGTACTGATCTGGCCTTTTACACCAACGGGCAG     1800
tetW-5       ATTCCCGCCCGCTGTATACAGGCATACCGTACTGATCTGGCCTTTTACACCAACGGGCAG     1800
tetW-1       ATTCCCGCCCGCTGTATACAGGCATACCGTACTGATCTGGCCTTTTACACCAACGGGCGG     1800
tetW-2       ATTCCCGCCCGCTGTATACAGGCATACCGTACTGATCTGGCCTTTTACACCAACGGGCAG     1800
             *********** ** ********************* ****** ********* **** *

tetW-3       AGCGTGTGCCTGACGGAACTGAAAGGGTATCAGGCCGCTGTCGGCCAGCCGGTCATCCAG     1860
tetW-4       AGCGTATGCCTTACAGAACTGAAAGGGTATCAGGCCGCTGTCGGCAAGCCAGTCATCCAG     1860
tetW-5       AGCGTATGCCTTACAGAACTGAAAGGGTATCAGGCCGCTGTCGGCAAGCCAGTCATCCAG     1860
tetW-1       AGCGTATGCCTTACAGAGCTGAAAGGATATCAGGCCGCTGTCGGTCAGCCGGTCATCCAG     1860
tetW-2       AGCGTATGCCTTACAGAGCTAAAAGGGTATCAGGCCGCTGTCGGCCAGCCGGTCATCCAG     1860
```

```
              ***** ***** ** ** ** ***** *****************  **** *********

tetW-3        CCCCGCCGTCCAAACAGCCGCCTGGACAGGGTGCGCCATATGTTTCAGAAGGTAATGTAA    1920
tetW-4        CCCCGCCGTCCAAACAGCCGCCTGGACAAGGTGCGCTATATGTTTCAGAAGATAATGTAA    1920
tetW-5        CCCCGCCGTCCAAACAGCCGCCTGGACAAGGTGCGCTATATGTTTCAGAAGATAATGTAA    1920
tetW-1        CCCCGCCGTCCAAACAGCCGCCTGGACAAGGTGCGCCATATGTTTCAGAAGGTAATGTAA    1920
tetW-2        CCCCGCCGTCCAAACAACCGCCTGGACAAGGTGCGCCATATGTTTCAGAAGGTAATGTAA    1920
              **************** *********** ******* ************** ********

tetW-3        CGTCTTGCGCAATACAAGCGTTCATTGCTGGCTATTGCGAAGTTTGTCGGATAAATAGCA    1980
tetW-4        ------------------------------------------------------------    1920
tetW-5        ------------------------------------------------------------    1920
tetW-1        AGATACATAATCGTCAAGACGGCAACAATCAGAAGTTAT------GGAGGGTAACA----    1970
tetW-2        ------------------------------------------------------------    1920
```

Figure S1. Multiple nucleotide sequence alignment of different variants of the *tetW* gene. Selected nucleotide sequences of the *tetW* variants were obtained from the GenBank database: *Butyrivibrio fibrisolvens tetW-1* (AJ427421.2), *Megasphaera elsdenii tetW-2* (AY485124.1), *Trueperella pyogenes tetW-3* (AY049983.2), *Trueperella pyogenes tetW-4* (DQ517519.1), *Trueperella pyogenes tetW-5* (DQ519395.1). The multiple alignment was done using Clustal Omega. The asterisk (*) shows identical residues whilst the break shows differences. The comparison includes the 1920-bp complete sequences of *tetW* and flanking regions of *tetW-1* and *tetW-3* downstream the genes (in bold). The positions of universal primers for genes encoding tetracycline resistance ribosomal protection proteins (DI-F: GAYACICCIGGICAYRTIGAYTT, DII-R: GCCCARWAIGGRTTIGGIGGIACYTC) are highlighted in blue. The positions of *tetW* primers designed by Billington and Jost (tetW_F: GACAACGAGAACGGACACTATG, tetW_R: CGCAATAGCCAGCAATGAACGC) [37] are highlighted in yellow. The positions of *tetW* primers designed in this study to detect all variants of *tetW* gene (tetW-all_F: GTCTGTTCGGGATAAGCTCT), tetW-all_R: TGGAATACGCATCTCTGTGA) are highlighted in green.
