# Supplementary material for: Trueperella pyogenes Isolates from Livestock and European Bison (Bison bonasus) as a Reservoir of Tetracycline Resistance Determinants
Source: Antibiotics (Basel). 2021 Apr 3;10(4):380. doi: 10.3390/antibiotics10040380 (PMC8065510; doi:10.3390/antibiotics10040380)
Supplement: Supplementary file 1 [file antibiotics-10-00380-s001.zip › antibiotics-1130529-supplementary/Supplementary Materials - Kwiecie¿╜ Ewelina/Table S1.pdf]

**Table S1.** Origin and characteristics of *Trueperella pyogenes* isolates ($n$=19) identified in this study, which have not been previously described.

| **Isolate designation**[a] | **Animal species** | **Source of isolate** | **Catalase production**[b] | **Detection of *plo***[c] |
|---|---|---|---|---|
| 32/B | cattle | mastitis | - | + |
| 33/B | cattle | mastitis | - | + |
| 34/B | cattle | mastitis | - | + |
| 35/B | cattle | mastitis | - | + |
| 36/B | cattle | mastitis | - | + |
| 37/B | cattle | mastitis | - | + |
| 38/B | cattle | mastitis | - | + |
| 49/S | swine | purulent lesion in lymph nodes | - | + |
| 914/Z | European bison | purulent lesion in trachea | - | + |
| 919/Z | European bison | purulent lesion in trachea | - | + |
| 237/Z | European bison | purulent vaginal discharge | - | + |
| 438/Z | European bison | purulent vaginal discharge | - | + |
| 1521/Z | European bison | purulent vaginal discharge | - | + |
| 14/K | goat | purulent vaginal discharge | - | + |
| 15/K | goat | purulent vaginal discharge | - | + |
| 20/K | goat | abscess of lungs | - | + |
| 27/K | goat | purulent vaginal discharge | - | + |
| 35/K | goat | abscess of lungs | - | + |
| 144/O | sheep | purulent lesion in lymph nodes | - | + |

[a] *B – bovine, S – swine, Z – European bison, K – caprine.*
[b] *-:a negative reaction.*
[c] *+: a presence of the gene.*
